# Prognostic Value of the Average Lung CT Number in Patients with Acute Paraquat Poisoning

**DOI:** 10.1155/2023/4443680

**Published:** 2023-09-12

**Authors:** Xinrui Jiang, Hengjun Liu, Geng Lu, Jiawei Zhou, Jun Wang, Binxia Shao, Peng Xu

**Affiliations:** Department of Emergency, Nanjing Drum Tower Hospital, Medical School of Nanjing University, Nanjing, China

## Abstract

**Objective:**

The chest computed tomography (CT) examination is an important clinical examination in the diagnosis and monitoring of paraquat- (PQ-) induced lung injury. The aim of this study was to explore the prognostic value of the average lung CT number acquired by quantitative CT techniques in patients with acute paraquat poisoning in the early stages of the disease.

**Methods:**

46 patients who suffered from acute PQ poisoning in the emergency department of the Nanjing Drum Tower Hospital from January 2015 to June 2020 were enrolled in the present study. The patients were divided into survival group (*n* = 21) and nonsurvival group (*n* = 25). Clinical data were collected from subjects who met the inclusion criteria, including general information, personal disease history, and laboratory test indicators. The average lung CT numbers of each patient were obtained by quantitative CT techniques. Receiver operating characteristic (ROC) analysis was conducted to assess the prognostic value of average lung CT number in patients with acute paraquat poisoning.

**Results:**

The average CT numbers of the middle-lung, lower-lung, and whole lung fields in the nonsurvival group were significantly higher than those of the survival group (*p* < 0.0001). However, the upper-lung field was not significantly different between the two groups (*p* = 0.7765). The AUCs of different levels ranged from 0.554 to 0.977, among which the lower-lung field presented the largest AUC of 0.977 (95% CI: 0.943∼1; cut-off value: −702Hu; sensitivity 96%; specificity, 90.5%; YI: 0.865), followed by the whole lung field 0.914 (95% CI: 0.830∼0.999; cut-off value: −727Hu; sensitivity 76%; specificity, 95.2%; YI: 0.712) and the middle-lung field 0.87 (95% CI: 0.768∼0.971; cut-off value: −779Hu; sensitivity 80%; specificity, 85.7%; YI: 0.657).

**Conclusion:**

The present study indicated that the average lung CT number could be used to evaluate the relationship between the severity of PQ-induced lung injury and prognosis, especially in the lower-lung field. However, further research is needed to draw a clear conclusion.

## 1. Introduction

Paraquat (PQ) is a widely used herbicide in agriculture. Despite its frequent use, it is highly toxic to both humans and animals [1]. Although certain Asian countries have implemented strategies to strictly control its sales and use, PQ poisoning incidents occur frequently, and the overall mortality rate is estimated to be higher than 50%, mainly due to the intrinsic toxicity of this compound and the lack of effective treatment methods [2]. The lethal dose in adults is very low. Specifically, only 5–15 ml 20% PQ aqueous solution can be lethal [3]. Following its absorption through the gastrointestinal tract, the plasma concentration of PQ reaches maximum levels from 0.5 to 4 h. During that period, this compound is rapidly distributed to the lungs, kidneys, liver, muscles, and other tissues, causing damage. The main target organ involved is the lung, in which the concentration of PQ is 10–90 times higher than that noted in plasma [4, 5].

Although several basic research and clinical research studies have been performed on PQ poisoning, the results have not been summarized by international guidelines regarding the prevention of this disease [6]. Regrettably, there is no specific antidote available and the patients are mainly treated with supportive treatment. For patients with severe poisoning, it is difficult to improve the prognosis even with various treatment methods, while for some patients with mild poisoning, reasonable management and treatment can obtain a good prognosis. Thus, the early prediction of the severity of acute PQ poisoning can aid the guidance of appropriate treatment. Current studies on the prognosis of paraquat poisoning mainly include the amount of paraquat ingested [7], hematological indicators [8–10], biological indicators [11–13], various rating scales [14, 15], and ultrasound and imaging indicators [16, 17]. However, no unified clinical standards are present for prognostic evaluation of PQ poisoning to date. Clinicians usually judge the severity of the disease according to the amount of paraquat ingested, the concentration of PQ in blood or urine, and other clinical examination findings. Besides, these markers have not been widely used in the majority of hospitals due to the requirement for specialized and expensive equipment. In addition, the clinical judgment of the intake dose of PQ poisoning is easily affected by factors, such as the patient's self-description, vomiting, gastric lavage, PQ dosage form, and other factors. It is often difficult for doctors to assess the progression of the disease based on the poisoning dose described by the patient.

Chest CT is an important method for the clinical diagnosis and monitoring of PQ-induced lung injury and has become a routine examination of PQ poisoning in hospitalized patients [18]. Numerous studies [17, 19–21] have demonstrated that chest CT is also of high value in the prognosis of PQ poisoning, while the optimal indicator remains unclear. Computed tomography (CT) number, also known as the Hounsfield Unit, is a relative quantitative measurement of x-ray density [22]. It is the most frequently used quantitative CT parameter and widely used in radiological diagnosis, attenuation correction, and radiotherapy treatment planning [23]. However, there are few clinical studies on the effect of the average lung CT number on the prognosis of acute PQ poisoning patients. Therefore, the purpose of this study was to analyze the predictive impact of the average lung CT number on the prognosis of acute PQ poisoning patients in order to explore whether it can be used as a prognostic index in acute PQ poisoning patients.

## 2. Methods and Materials

### 2.1. General Information

**Figure 1:**
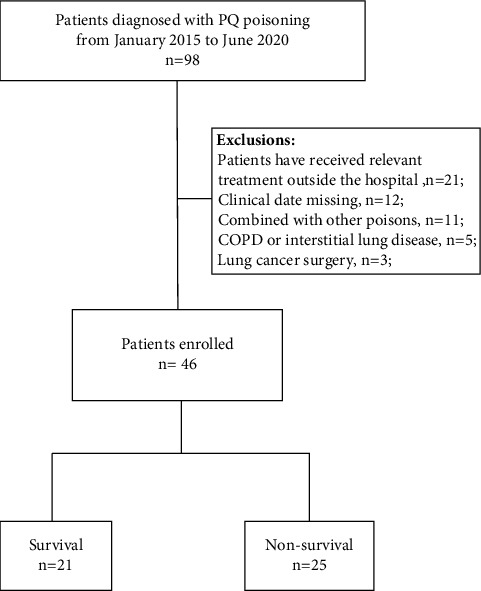
Flow chart indicating enrolment and status of patients.

The present retrospective analysis collected medical records of patients diagnosed with PQ poisoning from January 2015 to June 2020. The patient screening process is shown in Figure 1. Finally, 46 patients were included in the study (Table S1). According to the disease prognosis, the patients were divided into survival group (*n* = 21) and nonsurvival group (*n* = 25).

### 2.2. Inclusion and Exclusion Criteria

The inclusion criteria were as follows: (1) age higher than and/or equal to 16 years and lower than and/or equal to ≤75 years; (2) diagnostic criteria for acute PQ poisoning, such as relevant exposure history to toxic compounds; and (3) measurement of PQ concentration in plasma. Similar diseases caused by other causes were excluded.

The exclusion criteria were as follows: (1) patients who suffered acute or chronic lung disease; (2) already have received relevant treatment and chest CT examination outside the hospital; (3) other types of pesticide poisoning; (4) pregnancy; (5) cases with malignant tumors; and (6) missing clinical data.

## 3. Patients Management and Outcomes

Based on the experience obtained from the local diagnostic and treatment team and from previously published studies [2, 3], all enrolled patients received a corresponding therapeutic regimen during their hospitalization, including gastric lavage, hemoperfusion, hemofiltration, glucocorticoids, immunosuppressants, antioxidants, anti-infectives, and other symptomatic support medications. Data supplement presents the protocol for PQ detoxification.

### 3.1. Research Methods

The initial clinical baseline data of all patients were collected, including gender, age, mode of poisoning, oral dose of poisoning, time from poisoning to medical treatment, time from admission to chest CT examination, time from poisoning to chest CT examination, hospital length of stay, body temperature, pulse rate, respiratory rate, mean arterial pressure, finger pulse oxygen, smoking history, drinking history, and plasma concentration of paraquat.

**Figure 2:**
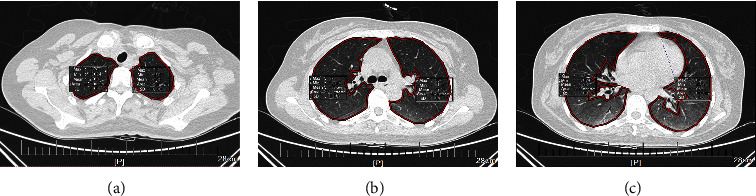
The 3 representative levels of chest HRCT. (a) Carinal plane, (b) 5 cm plane above carina, (c) 5 cm plane below carina.

The chest CT images of the patients on admission were collected. Chest CT examination of patients with acute PQ poisoning was routinely performed using the Philips ICT apparatus (128 rows) with a scanning layer thickness of 1-2 mm. The imaging data were analyzed using the Neusoft-picture archiving and communication system (PACS) (VE 5.5). The three standard levels of chest HRCT were defined as follows: carinal plane, 5 cm plane above the carina, and 5 cm plane below the carina (Figure 2) [24–26]. The average CT numbers of the three levels represent the mean lung density of the upper-, middle-, and lower-lung fields, respectively. The average CT number of the whole lung was defined as the average of three standard level measurements. The measurement tools in the PACS system were used to select the largest lung field area as far as possible, also known as the region of interests (ROIs). Subsequently, the average CT numbers (window level, −600 HU; window width, 1,600 HU) of the ROIs were calculated automatically. The same standard level was measured twice, and the average of the two measurements was obtained as the average CT number of the level.

### 3.2. Statistical Analysis

All analyses were conducted using SPSS version 22 (SPSS Inc., Chicago, IL, USA). The analysis of the measurement data was performed using an independent sample *t*-test. The analyzed data are presented as mean ± standard deviation. The count data are expressed as a number of cases (percentage), and the comparison between the groups was performed by the *χ*^2^ test. The ROC curves were computed, and the areas under the curves (AUCs) were used to study the discriminatory power of the lung average CT number regarding mortality. The parameters sensitivity, specificity, Youden index (YI), and cut-off value (sensitivity + specificity-1) of the various predictors were also calculated to provide a complete description of the prediction parameters. A two-tailed *p* <  0.05 was considered to indicate a statistically significant difference.

## 4. Results

### 4.1. Baseline Characteristics

The present study enrolled 98 patients with acute PQ poisoning, of whom 52 did not meet the eligibility criteria and were excluded (Figure 2). The final analysis included 46 patients, of whom 25 comprised the nonsurvival group (12 males/13 females; mean age 44.68 ± 17.026 years) and 21 the survival group (10 males/11 females; mean age 40.52 ± 14.327 years). No differences were noted in age, sex ratio, mode of poisoning, time from poisoning to treatment, time from admission to chest CT examination, time from poisoning to chest CT examination, hospital length of stay, body temperature, respiratory rate, heart rate, respiratory rate, mean arterial pressure and finger pulse oxygen, rate of smokers, and rate of drinkers between the survivor and nonsurvival groups (all *p* > 0.05). It is expected that the toxic dose described by the patients correlated with the plasma concentration of PQ. The differences were statistically significant between the two groups (*p* < 0.01) (Table 1).

### 4.2. Comparison of the Average CT Number between Two Groups

The average CT numbers of the middle-lung, lower-lung and whole lung fields in the nonsurvival group were significantly higher than those of the survival group (*p* < 0.0001). However, the upper-lung field was not significantly different between the two groups (*p*=0.7765) (Table 2).

### 4.3. ROC Curve Analysis

**Figure 3:**
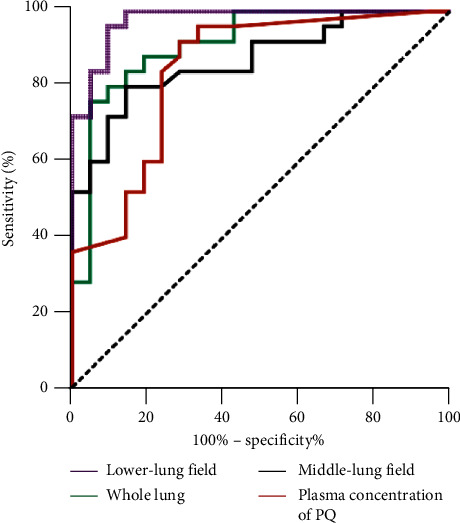
Comparison of receiver operating characteristics curves of the middle-lung field, lower-lung field, whole lung and the plasma concentration of PQ for predicting the outcome of acute PQ poisoning.

To further evaluate the predictive value of the average CT number of the lung tissue, we performed ROC curve analysis on the average CT number of different levels and of the whole lung. The AUCs, cut-off value, sensitivity, specificity, and YI were calculated. The AUCs of different levels ranged from 0.554 to 0.977 (Figure S1). In particular, the lower-lung field presented the largest AUC of 0.977 (95% CI: 0.943∼1; cut-off value: −702Hu; sensitivity: 96%; specificity, 90.5%; YI: 0.865), followed by the whole lung 0.914 (95% CI: 0.830∼0.999; cut-off value: −727Hu; sensitivity: 76%; specificity, 95.2%; YI: 0.712) and the middle-lung field 0.87 (95% CI: 0.768∼0.971; cut-off value: −779; sensitivity: 80%; specificity, 85.7%; YI: 0.657). Similar results were noted for the carinal plane (right) and the right and left sides of the plane 5 cm below the carina (Figure 3 and Table 3).

## 5. Discussion

Acute PQ poisoning can cause multiple organ dysfunctions, such as lung injury, acute kidney injury (AKI), and liver dysfunction. Although the morbidity rate of this disease has decreased compared with previous years, its mortality rate remains high [6]. Patients with acute PQ poisoning are vulnerable and exhibit a low healing rate. Although the measurement of blood or urine PQ concentration is currently a reliable predictor, it is not easily available in all hospitals. In clinical practice, it is also difficult for doctors to accurately determine the dose of poison (Table S1). So far, the evaluation criteria for the prognosis of acute PQ poisoning have not yet been clinically unified. In the present study, the relationship between the average lung CT number and the prognosis in the early stage of PQ poisoning was explored. Our results indicated that the average lung CT numbers in the lower-lung, middle-lung, and whole lung fields were significantly different between the survival group and the nonsurvival group. By using further analysis, it was shown that the average lung CT number was a good predictor of mortality in patients with acute PQ poisoning, especially in the lower-lung field.

Lung injury caused by PQ is one of the most common clinical manifestations of this condition. The mechanism of lung injury caused by PQ has not yet been fully elucidated and mainly includes acute inflammatory responses, free radical production, oxidative stress, lipid peroxidation, abnormal gene response, and activation of various signaling pathways [2, 27–29]. PQ-induced lung injury is a dynamic process of pathological evolution [20]. In 1991, Im et al. [30] retrospectively analyzed the characteristics of chest CT changes in 45 patients with acute PQ poisoning and confirmed this dynamic evolutionary process. Similar results were noted in a rat model of acute PQ poisoning [31]. Chest CT examination, notably high-resolution CT, is an important method to diagnose and monitor PQ-induced lung injury and has been widely used in hospitals. Lung CT findings can be used as a prognostic indicator for patients with lung injury caused by acute PQ poisoning. In 1994, Lee et al. [20] demonstrated that ground glass changes on chest imaging exhibited prognostic value in patients with acute PQ poisoning. Zhang et al. [17] compared the chest CT findings of 78 patients with acute PQ poisoning following admission and 3 days following treatment. The results indicated that the patients in the death group exhibited a higher number of lung lesions and a higher incidence of exudation, consolidation, and fibrosis compared with those of the survival group. The conduct of multiple chest CT examinations during treatment can assess the evolution of lung injury in a timely fashion and provide important information for improving clinical treatment. However, the evaluation indicators and clinical criteria of chest CT in patients with acute early-stage PQ poisoning remain to be improved. Kang et al. [32] quantitatively analyzed the lungs of 97 cases of PQ poisoning within 5 days, and the results indicated that the ground-glass opacity volume ratio could be used as a new, reliable, and independent indicator for predicting the outcome of acute PQ poisoning. This index was superior to the estimated PQ intake, blood or urine PQ concentration, Acute Physiology and Chronic Health Evaluation II (APACHE-II), and Sequential Organ Failure Assessment Score (SOFA). The optimal cut-off value of the GGO volume ratio was 10.8%, while the sensitivity and specificity were 85.4% and 89.3%, respectively. Liu et al. [33] prospectively evaluated the prognostic roles of quantitative CT and pulmonary function tests and the association of dynamic strain and ventilation heterogeneity during unassisted spontaneous breathing with 90-day survival in 100 patients with PQ poisoning. The data of this study indicated that Exp (%Wpoor/15) and the percentage of residual volume/total lung capacity were independent prognostic indicators. In addition, higher dynamic strain and increased ventilation heterogeneity during unassisted spontaneous breathing were associated with reduced survival independent of these two indicators, which suggested that the role of lung strain changes could not be ignored in disease prognosis following PQ poisoning. Furthermore, Liu et al. [34] proposed that the ratio of damaged lung volume fraction could also be used to evaluate the prognosis of acute PQ poisoning by combining the region growing segmentation method and an artificial neural network. However, the clinical operability was not appropriate.

In the present study, the prognostic value of the average lung CT number was evaluated in patients with acute PQ poisoning. The data indicated that the diagnostic power of the average CT number in lower-lung, middle-lung, and whole lung fields were superior to that of the plasma PQ concentration, which was a univariate predictor of the prognosis of patients with acute PQ poisoning. It is worth noting that the average lung CT numbers in different levels consist with the lung injury. The average CT number in the lower-lung field was higher than the middle- and upper-lung fields no matter in the nonsurvival group and the survival group, which was probably attributed to the gravitational force.

The present study contains several limitations. First, our sample size was small, mainly due to the government's strict control over the sales policy and the use of PQ. Second, only 3 representative levels were selected for the present study, which could not completely simulate the changes in the average whole lung CT number. Finally, we did not evaluate the association between a dynamic change in the average CT number and the prognosis of patients with acute PQ poisoning.

## 6. Conclusion

In the present study, we demonstrated that the average lung CT number was an optimal early predictor of mortality in patients with acute PQ poisoning, especially in the lower-lung field. However, further research is required to confirm this finding.

## Figures and Tables

**Table 1:** Comparisons of baseline characteristics between the survival and nonsurvival groups with acute PQ poisoning.

Parameters	Survival (*n* = 21)	Nonsurvival (*n* = 25)	*p* value (two tailed)
Age	40.52 ± 14.327	44.68 ± 17.026	0.381
Sex (female)	11 (52.38%)	13 (52%)	0.980
Mode of poisoning (suicide)	15 (71.43%)	23 (92%)	0.067
Time from poisoning to treatment (h)	8.17 ± 5.223	7.60 ± 3.452	0.6477
Time from admission to chest CT examination (d)	2.563 ± 1.482	2.125 ± 1.221	0.278
Time from poisoning to chest CT examination (h)	69.69 ± 34.7608	58.6 ± 28.9036	0.244
Hospital length of stay	7.19 ± 0.836	4.76 ± 5.464	0.094
Body temperature (°C)	36.676 ± 0.3177	36.884 ± 0.5367	0.112
Pulse rate (beats/min)	77.476 ± 15.4907	82.68 ± 14.1354	0.240
Respiratory rate (breaths/min)	18.857 ± 1.9049	20.04 ± 2.5736	0.088
Mean arterial pressure (mmHg)	94.698 ± 13.2106	96.333 ± 12.6652	0.671
Finger pulse oxygen	95.962 ± 3.6757	96.48 ± 3.1901	0.6611
Smoking history^*∗*^	2	3	0.794
Drinking history^*∗*^	1	2	0.666
Toxic dose (ml)	19.3 ± 15.3	35.4 ± 17.9	<0.0001
Plasma concentration of paraquat (*μ*g/ml)^†^	0.444 ± 0.805	3.906 ± 5.226	0.0031

^*∗*^Two patients in the nonsurvival group had both a history of smoking and drinking, while one patient had a history of both smoking and drinking in the survival group. ^†^Plasma concentration of paraquat was detected by high performance liquid chromatography (HPLC).

**Table 2:** Comparison of lung average CT number at different levels between the survival and nonsurvival groups with acute PQ poisoning ($\bar{x}\pm s$).

Parameters	Survival (*n* = 21)	Nonsurvival (*n* = 25)	*p* value
Upper-lung field (Hu)	−766.833 ± 52.372	−771 ± 44.980	0.7765
Middle-lung field (Hu)	−797.381 ± 27.569	−730.1 ± 59.012	<0.0001
Lower-lung field (Hu)	−761.714 ± 50.743	−610.32 ± 56.014	<0.0001
Whole lung field (Hu)	−775.310 ± 39.448	−703.807 ± 40.943	<0.0001

**Table 3:** Predictive value of various indicators for the prognosis of patients with acute PQ poisoning.

Parameters	Cut-off	Sensitivity (%)	Specificity (%)	YI	AUC	95% CI
Middle-lung field (Hu)	−779	80	85.7	0.657	0.87	0.768∼0.971
Lower-lung field (Hu)	−702	96	90.5	0.865	0.977	0.943∼1
Whole lung (Hu)	−727	76	95.2	0.712	0.914	0.830∼0.999
Plasma concentration of paraquat (*μ*g/ml)	0.189	92	71.4	0.634	0.845	0.728∼0.962

## Data Availability

All data generated or analyzed during this study are included in this published article. Raw and processed data are available upon request.

## Supplementary Materials

Supplementary Materials Protocol for PQ detoxification. Table s1. Basic information of included patients. Figure S1. ROC analysis of different levels.
