# Supplementary material for: Prognostic Value of the Average Lung CT Number in Patients with Acute Paraquat Poisoning
Source: Emerg Med Int. 2023 Sep 12;2023:4443680. doi: 10.1155/2023/4443680 (PMC10508996; doi:10.1155/2023/4443680)
Supplement: Supplementary Materials — Protocol for PQ detoxification. Table s1. Basic information of included patients. Figure S1. ROC analysis of different levels. [file 4443680.f1.zip › Protocol for PQ detoxification (1).docx]

Protocol for PQ Detoxification

一、Reduction of toxic absorption

1. Terminate paraquat exposure, including removal of contaminated clothing and skin rinsing.

2.Vomiting and gastric lavage: It is usually used within 6 hours after paraquat poisoning; however, for patients with high gastric emptying or ingestion, gastric lavage can still be considered after 6 hours. We usually wash the stomach with warm saline, using low pressure for repeated flushes, totaling about 5 L, until it is colorless and odorless.

二、Blood Purification

We usually use HP in combination with CVVH, after which the follow-up protocol is decided according to the patient's toxic dose and renal function.

三、Medication regimens

These include glucocorticoids, immunosuppressants, antioxidants, anti-infectives and other symptomatic support medications.

1. Anti-inflammatory treatment: in patients with mild disease, we usually use corticosteroids alone, while in patients with moderate to severe disease, we usually combine glucocorticoids and cyclophosphamide for anti-inflammatory therapy. The initial dose of methylprednisolone is 3-15 mg/(kg🞌d) or equivalent dose of other glucocorticoids, and the dose of cyclophosphamide ranges from 2-15 mg/(kg🞌d), which is usually tapered after 3 d of application. The initial dose and the magnitude and method of taper are determined by the patient's clinical presentation and pulmonary imaging and immune status.

2.Antioxidant therapy: including vitamin C and N-acetylcysteine.

3. Anti-fibrotic drugs are recommended, but not available in our center.

4.Other symptomatic support medications: including protection of gastrointestinal mucosa, hepatoprotective therapy and rehydration and diuresis etc.

四、Other treatments

Oxygen therapy and mechanical ventilation strategies: r outine oxygen administration should be avoided in the early stages in patients with paraquat poisoning. For patients with respiratory failure we will evaluate mechanical ventilation and ECMO indications.
