# Supplementary material for: Prognostic Value of the Average Lung CT Number in Patients with Acute Paraquat Poisoning
Source: Emerg Med Int. 2023 Sep 12;2023:4443680. doi: 10.1155/2023/4443680 (PMC10508996; doi:10.1155/2023/4443680)
Supplement: Supplementary Materials — Protocol for PQ detoxification. Table s1. Basic information of included patients. Figure S1. ROC analysis of different levels. [file 4443680.f1.zip › Supplementary Materials.docx]

**Supplementary Materials**

Protocol for PQ detoxification.

**Figure S1. ROC analysis of different levels.**
