# Supplementary material for: Prognostic Value of the Average Lung CT Number in Patients with Acute Paraquat Poisoning
Source: Emerg Med Int. 2023 Sep 12;2023:4443680. doi: 10.1155/2023/4443680 (PMC10508996; doi:10.1155/2023/4443680)
Supplement: Supplementary Materials — Protocol for PQ detoxification. Table s1. Basic information of included patients. Figure S1. ROC analysis of different levels. [file 4443680.f1.zip › Table s1. Basic information of included patients.docx]

**Table s1. Basic information of included patients.**

| **Case No.** | **Sex** | **Age** | **Mode of poisoning** | **Time from poisoning to treatment (h)** | **Toxic dose(ml)** | | **Survival** |
| --- | --- | --- | --- | --- | --- | --- | --- |
| 1 | Male | 53 | Suicide | 10 | 50 | | Dead |
| 2 | Male | 32 | Suicide | 8 | 200 | | Dead |
| 3 | Female | 28 | Suicide | 5 | 40 | | Dead |
| 4 | Male | 75 | Misuse | 10 | 15 | | Dead |
| 5 | Female | 39 | Suicide | 13 | 30 | | Dead |
| 6 | Female | 27 | Suicide | 16 | 40 | | Dead |
| 7 | Male | 23 | Suicide | 9 | 50 | | Dead |
| 8 | Female | 51 | Suicide | 4 | 50 | | Dead |
| 9 | Male | 20 | Suicide | 7 | 100 | | Dead |
| 10 | Male | 57 | Suicide | 8 | 200 | | Dead |
| 11 | Female | 32 | Suicide | 6 | 30 | | Dead |
| 12 | Female | 49 | Suicide | 16 | 50 | | Dead |
| 13 | Male | 69 | Suicide | 5 | 100 | | Dead |
| 14 | Female | 72 | Suicide | 3 | 100 | | Dead |
| 15 | Male | 64 | Suicide | 5 | 60 | | Dead |
| 16 | Male | 30 | Suicide | 5 | 20 | | Dead |
| 17 | Male | 28 | Suicide | 6 | 60 | | Dead |
| 18 | Female | 50 | Suicide | 11 | 50 | | Dead |
| 19 | Female | 58 | Suicide | 6 | 100 | | Dead |
| 20 | Female | 48 | Misuse | 7 | 20 | | Dead |
| 21 | Female | 33 | Suicide | 7 | 20 | | Dead |
| 22 | Male | 51 | Suicide | 7 | 50 | | Dead |
| 23 | Female | 39 | Suicide | 5 | 50 | | Dead |
| 24 | Female | 68 | Suicide | 7 | 100 | | Dead |
| 25 | Male | 21 | Suicide | 4 | 50 | | Dead |
| 26 | Male | 30 | Suicide | 4 | 15 | | Alive |
| 27 | Male | 25 | Misuse | 7 | 5 | | Alive |
| 28 | Male | 43 | Suicide | 6 | 15 | | Alive |
| 29 | Male | 29 | Suicide | 5 | 10 | | Alive |
| 30 | Male | 51 | Suicide | 8 | 50 | | Alive |
| 31 | Female | 49 | Suicide | 5 | 15 | | Alive |
| 32 | Female | 56 | Misuse | 8 | 5 | | Alive |
| 33 | Female | 33 | Misuse | 3 | 3 | | Alive |
| 34 | Female | 58 | Suicide | 3 | 20 | | Alive |
| 35 | Male | 71 | Misuse | 12 | 5 | | Alive |
| 36 | Female | 34 | Suicide | 6 | 10 | | Alive |
| 37 | Male | 50 | Suicide | 5 | 15 | | Alive |
| 38 | Male | 24 | Misuse | 5 | 3 | | Alive |
| 39 | Female | 49 | Suicide | 5 | 30 | | Alive |
| 40 | Female | 24 | Suicide | 8 | | 50 | Alive |
| 41 | Female | 49 | Suicide | 10 | | 5 | Alive |
| 42 | Male | 33 | Suicide | 24 | | 5 | Alive |
| 43 | Female | 48 | Suicide | 11 | | 15 | Alive |
| 44 | Male | 14 | Misuse | 16 | | 3 | Alive |
| 45 | Female | 29 | Suicide | 5 | | 10 | Alive |
| 46 | Female | 52 | Suicide | 16 | | 5 | Alive |

***: The toxic dose was mainly obtained from the medical record based on the patient's self-report, but the exact dosage form and expiration date cannot be reached.**
